# Prevalence and correlates of generalized anxiety disorder and perceived stress among Sudanese medical students

**DOI:** 10.1186/s12888-024-05510-y

**Published:** 2024-01-23

**Authors:** Danya Ibrahim, Reem Mohamed Ahmed, Ayman Zuhair Mohammad, Basil Ibrahim, Tibyan Mohammed, Mona Elfadl Mohamed, Tibyan Abdelgadir, Baraah Mohammed, Moneib Ibrahim, Kamil M. A. Shaaban

**Affiliations:** 1 Faculty of Medicine, University of Khartoum, 11115 Khartoum, P. O Box: 321, Sudan; 2 Department of Psychiatry, Faculty of Medicine, University of Khartoum, Khartoum, Sudan; 3 Department of Surgery, Faculty of Medicine, University of Khartoum, Khartoum, Sudan; 4 Department of Community Medicine, Faculty of Medicine, University of Khartoum, Khartoum, Sudan

**Keywords:** Generalized anxiety disorder, Perceived stress, Lifestyle, Academic performance, Medical students

## Abstract

**Background:**

Generalized Anxiety Disorder (GAD) causes significant disturbance in an individual’s well-being and activity. Whereby, interfering with the dynamic progress in life. Also, anxiety is a product of stress and a major predictor of academic performance. This study aimed to assess the prevalence of Generalized Anxiety Disorder (GAD), measure levels of anxiety and perceived stress, evaluate the academic profile, identify lifestyle characteristics, and explore the relationship between these factors.

**Methods:**

In this cross-sectional study, 340 Sudanese medical students filled out online questionnaires, composed of the sociodemographic and lifestyle characteristics, academic profile, Generalized Anxiety Disorder-2 scale (GAD-2), and Perceived Stress Scale-10 (PSS-10). Descriptive and inferential statistics were applied using Statistical Package for Social Science (SPSS) Version 20.0 for data analysis.

**Results:**

Of 340 medical students, 3.8% of them were diagnosed with GAD, while 29.1% scored ≥ 3 in GAD-2, indicating a possible diagnosis. The study found that 9.7% of the participants used addictive substances, with 42% of them having high GAD-2 scores. Moreover, high anxiety levels were associated with high-stress scores (*p*-value = 0.000). Also, high GAD-2 scores were significantly associated with students who spent less than 10,000 SDG (18 USD) weekly, spent more time on entertainment using smart devices (*p*-value = 0.004), and had an unhealthy diet (*p*-value = 0.004). Low anxiety levels were associated with better sleep quality (*p*-value = 0.00), satisfaction with religious practices (*p*-value = 0.00), and increased leisure/hobby time (*p*-value = 0.018). High-stress levels were observed in females (*p*-value = 0.035), those with lower academic performance satisfaction levels, and increased hours of smart device usage for entertainment (*p*-value = 0.001). Reduced stress levels were associated with being ≥ 23 years old, increased leisure/hobby time (*p*-value = 0.002), satisfaction with religious practices [F(3, 166.6) = 10.8, *p*-value = 0.00)], and having a healthy diet (*p*-value = 0.006).

**Conclusion:**

The low prevalence of GAD corresponded with previous literature, but 29.1% of medical students had a high probability of having GAD. The study emphasizes on providing accessible mental health services for medical students and interventions addressing modifiable risk factors.

## Background

***Generalized anxiety disorder*** **(GAD)** is a common and quite disabling psychiatric illness that often goes undiagnosed and untreated [1]. In a study conducted in the United States between (1980 and 1985), the ECA (Epidemiological Catchment Area) discovered that some anxiety problems were more common than other mood disorders, substance use disorders, and impulsive control problems [2, 3]. It is the most common anxiety disorder seen in primary care, affecting approximately 4–7% of U.S. adults [4, 5] and can affect up to 6% of people in their lifetime. If untreated, GAD has a chronic course and places a heavy social and financial strain [5].

**Fig. 1:**
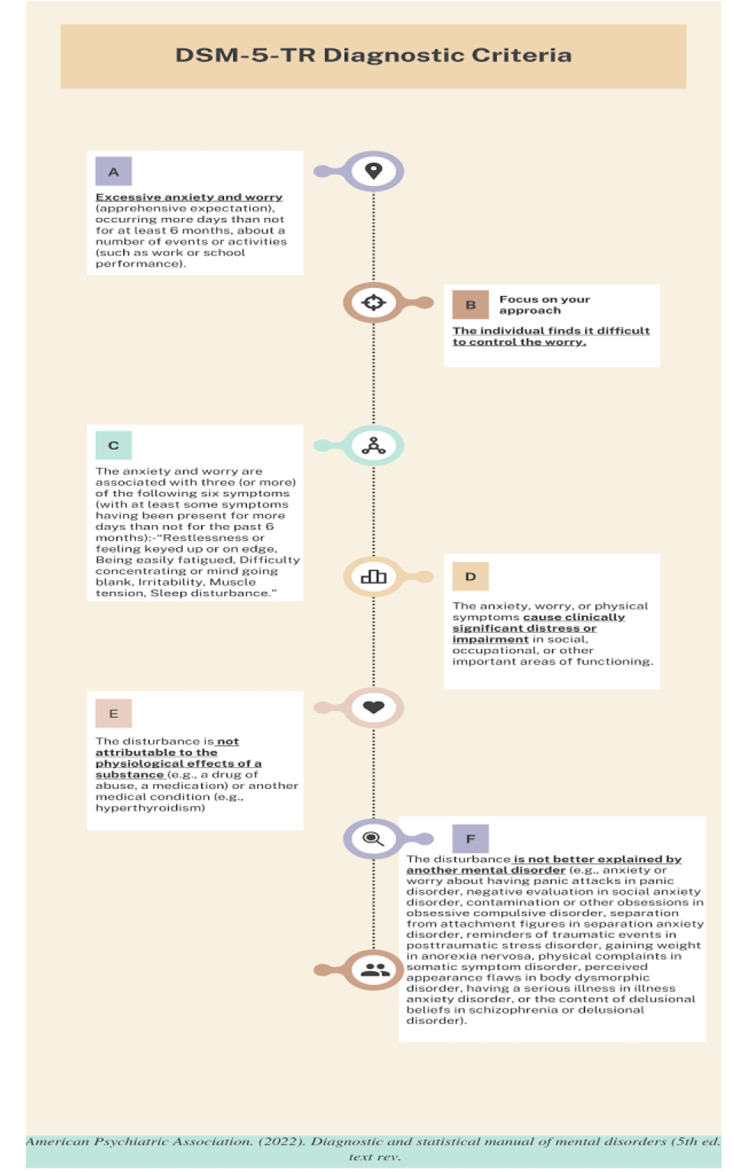
Shows DSM 5-TR diagnostic criteria for Generalized anxiety disorder (GAD)

Symptoms of GAD include chronic pervasive anxiety, restlessness, fatigue, difficulty concentrating, irritability, muscle tension, and sleep disturbances. The Diagnostic and Statistical Manual of Mental Disorders, Fifth Edition-Text Revision (DSM-5-TR), establishes GAD diagnosis based on the diagnostic criteria shown in Fig. 1.

Estimates of the lifetime prevalence of GAD according to the DSM-IV range from 0.1% in Nigeria to 6.2% in New Zealand highlighting the regional disparities in the prevalence of anxiety disorders, but the estimates are highly unclear and the current global prevalence of anxiety disorders is 7.3% (4.8–10.9%) [2].

Stress is any challenge to stability, or to the body’s internal sense of balance [6]. But, perceived stress is the feelings or thoughts that an individual has about how much stress they are under at a given point in time or over a given period [6].

A study conducted in Turkey among university students concluded that the prevalence of anxiety and stress were 47.1 and 27% respectively [7], while another study showed that a high prevalence of anxiety is present among the Saudi general population, the highest among younger participants, and students. However, the prevalence and severity of anxiety were higher in patients with chronic conditions and depressed patients [8]. Concerning medical students, the prevalence of perceived stress is high among medical students, especially females [9].

A study in Karachi, Pakistan showed high mental distress among medical students and it was mainly related to academic pressure. Moreover, the prevalence of anxiety and stress was 72% and 35% respectively. Students complained of poor lifestyles, lack of support services for stress management, and lack of physical and recreational facilities [10]. It seems that academic-related problems are greater perceived stressors. Perceived stress was statistically and strongly correlated with anxiety levels [11, 12].

High levels of perceived stress are strongly correlated to poor sleep hygiene [13]. Likewise, a study on university students concluded that students having higher perceived stress levels and comorbid insomnia were also likely to have higher anxiety levels [14]. Also, perceived stress and anxiety were significantly associated with academic performance [15]. Since medical schools are considered stressful settings for students, it has been hypothesized that there are more psychiatric illnesses among medical students [2].

Among the most prevalent mental health issues among medical students are anxiety and depression, which are also linked to low quality of life, disability, and poor academic performance. Furthermore, research indicated that the existing educational system may unintentionally have a detrimental impact on students’ mental health, with medical students being particularly susceptible to melancholy, anxiety, and stress. In addition, it has been suggested that medical school may be the source of burnout, a type of distress that is widespread among residents and practicing doctors [16].

The stress perceived by students can negatively affect their academic achievement, personal well-being, and long-term professional capabilities. Undergraduate students need to cope with the academic and social demands of university studies along their career pathway [17].

Students with anxiety disorders were found to display a passive attitude in their studies such as a lack of interest in learning, and poor performance in exams, and on assignments. Students with high levels of anxiety are unable to perform to the best of their ability.

Anxiety disorders, although as common and arguably as debilitating as depression, have garnered less attention, and are often undetected and under-treated in the general population. Similarly, anxiety among medical students warrants greater attention due to its significant implications. In February 2019, a systematic search for cross-sectional studies found that the global prevalence rate of anxiety among medical students was 33.8% (95% Confidence Interval: 29.2– 38.7%). Anxiety was most prevalent among Asian medical students. About one in three medical students globally have anxiety [18]. A cross-sectional study of medical students at Khartoum University, Sudan concluded that anxiety and stress prevalence was 22%, and 16% respectively [19]. Quality of environment and physical health were the major determinants of anxiety and stress.

Standing on previous studies GAD has received particularly little attention outside of a small handful of wealthy, developed nations, where almost all research on the illness has been done. But there is reason to believe that GAD may matter everywhere in the world as well, not just in wealthy nations. Given the greater economic and political instability, lack of access to necessities, and future uncertainty that is often present in lower-income nations, it is possible that GAD is more prevalent and detrimental there [20]. One of the major constraints to implementing adequate policy responses that promote and optimize mental health and address Generalized Anxiety Disorder and perceived stress in Sudan is the remarkable paucity of knowledge on the subject in the first place. To the best of our knowledge, there is extremely little data on Generalized Anxiety Disorder in Sudan, and nearly no data on the prevalence, correlates, and precipitants of the disorder among Sudanese medical students. More data on the prevalence of Generalized Anxiety Disorder, levels of perceived stress, and their relationship with sociodemographic characteristics, academic performance, and lifestyle can assist in understanding the issue and advocating for appropriate remedies, It may also be used as a baseline to help decision-makers at medical colleges and universities conduct programs and projects targeted at raising awareness, preventing and responding with such conditions, and improving the general mental health of medical students in Sudan. This study aims to explore the impact of stress in developing GAD and its detriment to academic performance and lifestyle among medical students in Khartoum University, Sudan, and to specifically: Assess the prevalence of generalized anxiety disorder among medical students, measure the level of perceived stress of each participant, investigate the relationship between anxiety level, perceived stress, academic profile, and lifestyle, and to investigate the relationship between perceived stress, academic profile, and lifestyle as well.

## Materials and methods

### Study design, setting, and study population

This is a descriptive cross-sectional study that was conducted at the University of Khartoum, Faculty of Medicine in October 2022.

The University of Khartoum is ranked 1st in Sudan and 2310th in the World 2022 overall rankings, plus got TOP 50% in 21 academic topics. Edu Rank’s ranking for the University of Khartoum is based on 3 factors: research performance (a proprietary algorithm evaluated 5,830 publications and 96,433 citations), non-academic reputation, and the impact of 36 notable alumni [21]. A total of 2325 medical students were registered in 2022 at the University of Khartoum. Medical students of both genders and from all academic levels were included in this study.

### Sampling strategy

The sample size was 342 students, calculated using population equation n = N / (1 + N e^2^) provided n = sample number, N = the population size (2325), e = the level of precision (0.05).

A systematic random sampling technique was used to select students from each batch, according to population proportion. A 10% non-response rate was added, and the final sample size was 340 students.

### Data collection tools

A self-administered anonymous online questionnaire designed with Google Forms, sent via telegram and WhatsApp was filled by 340 medical students with a response rate of 99.4%. The questionnaire consisted of five components: Sociodemographic criteria (e.g., age, gender, marital status, weekly expenditure, source of income), GAD-related questions including GAD-2 Scale, Perceived Stress Scale (PSS-10), academic performance (mean academic score and the level of satisfaction with the current academic performance), ending with lifestyle factors (e.g., smoking, energy drinks, physical exercise, eating habits) [22].

### Generalized anxiety disorder − 2 (GAD-2)

Consists of two items that work as a screening tool for generalized anxiety disorder. GAD-2 score is obtained by adding a score for each question. Anxiety Severity is calculated by assigning scores of 0, 1, 2, and 3 to the response categories, respectively, of “not at all”, “several days”, “more than half the days”, and “nearly every day”.

A score of 3 points is the preferred cut-off for identifying possible cases in which further diagnostic evaluation for generalized anxiety disorder is warranted. Using a cut-off of 3, the GAD-2 has a sensitivity of 86% and specificity of 83% for the diagnosis of generalized anxiety disorder [35]. The GAD-2 was based on the GAD-7, which has an excellent internal consistency of the GAD-7 (Cronbach α = 0.92) and good test-retest reliability (intraclass correlation = 0.83) [23]. The GAD-7 was developed by Drs. Robert L. Spitzer, Janet B.W. Williams, Kurt Kroenke, and colleagues based on the Primary Care Evaluation of Mental Disorders Patient Health Questionnaire (PRIME-MD-PHQ) [23]. With an educational grant from Pfizer Inc., no permission is required to reproduce, translate, display, or distribute.

### Perceived stress scale (PSS- 10)

The Perceived Stress Scale (PSS-10) is a 10-item questionnaire originally developed by Cohen et al. (1983) and widely used to assess stress levels in young people and adults aged 12 and above. It evaluates the degree to which an individual has perceived life as unpredictable, uncontrollable, and overloading over the previous month, with proven validity and reliability [24, 25]. The PSS-10 can be used by children aged 12 and above. The measure has been validated in both adolescent and adult populations. Initial evidence suggests that the PSS-10 may allow for meaningful comparisons across different racial, ethnic, or linguistic groups. Consisting of 10 questions that ask about feelings and thoughts during the last month. In each case, respondents are asked how often they felt a certain way on a Likert scale from ‘never’ to ‘very often’. Answers are then scored as follows: Never = 0, Almost never = 1, Sometimes = 2, fairly often = 3, Very often = 4. To calculate a total PSS score, responses to the four positively stated items (items 4, 5, 7, and 8) first need to be reversed (i.e., 0 = > 4; 1 = > 3; 2 = > 2; 3 = > 1; 4 = > 0). The PSS score is then obtained by summing across all items. The scores of 0–13 are considered mild, 14–26 are considered moderate and 27–40 scores indicate higher levels of perceived stress [26]. The Perceived Stress Scale is a screening and not a diagnostic instrument.

### Academic performance

The Faculty of Medicine at the University of Khartoum does not offer a grade point average GPA in its system but rather an individual grading for each subject in the final examination as follows: (Distinction = 80–100, very good = 70–79, good = 60–69, pass = 50–59, fail = 49 and less). Each subject grade was answered by one of the following: Distinction, Very good, Good, Pass, Fail, Not yet. A mean academic score was calculated for each student based on a scoring system the university uses to grade applicants for academic jobs in which distinction was given 4 points, very good was given 3 points, Good was given 2 points, a pass was given 1 point, and Fail was considered as zero. The mean academic score was then calculated by dividing the total score by the number of subjects the student had the result for. The mean academic score of all the students was calculated after.

### Data analysis

Students were divided into preclinical (first and second year) and clinical medical students (third, fourth, fifth, and sixth year). Data were extracted by the researchers from Google Forms into an Excel file. After re-coding the variables, data were transferred to Statistical Package for the Social Sciences (SPSS) version 20.0 for analysis. For descriptive statistics, mean and standard deviation were used for the normally distributed data. To assess the correlation between the GAD and the participants’ perceived stress and mean academic score, Spearman’s rho test was used. The Pearson correlation test was used to explore the correlation between perceived stress scores and participants’ Mean academic scores. The association between GAD score and academic performance satisfaction, demographic variables, and lifestyle factors was assessed using the Kruskal-Wallis test. For the association between Perceived stress and demographic variables, academic performance satisfaction, and lifestyle factors, the One-Way analysis of variance (ANOVA) test was used. ANOVA was also used to assess the association between the mean academic score and academic performance satisfaction. *P*-values of < 0.05 were considered statistically significant.

## Results

### Sociodemographic data of the participants

**Fig. 2:**
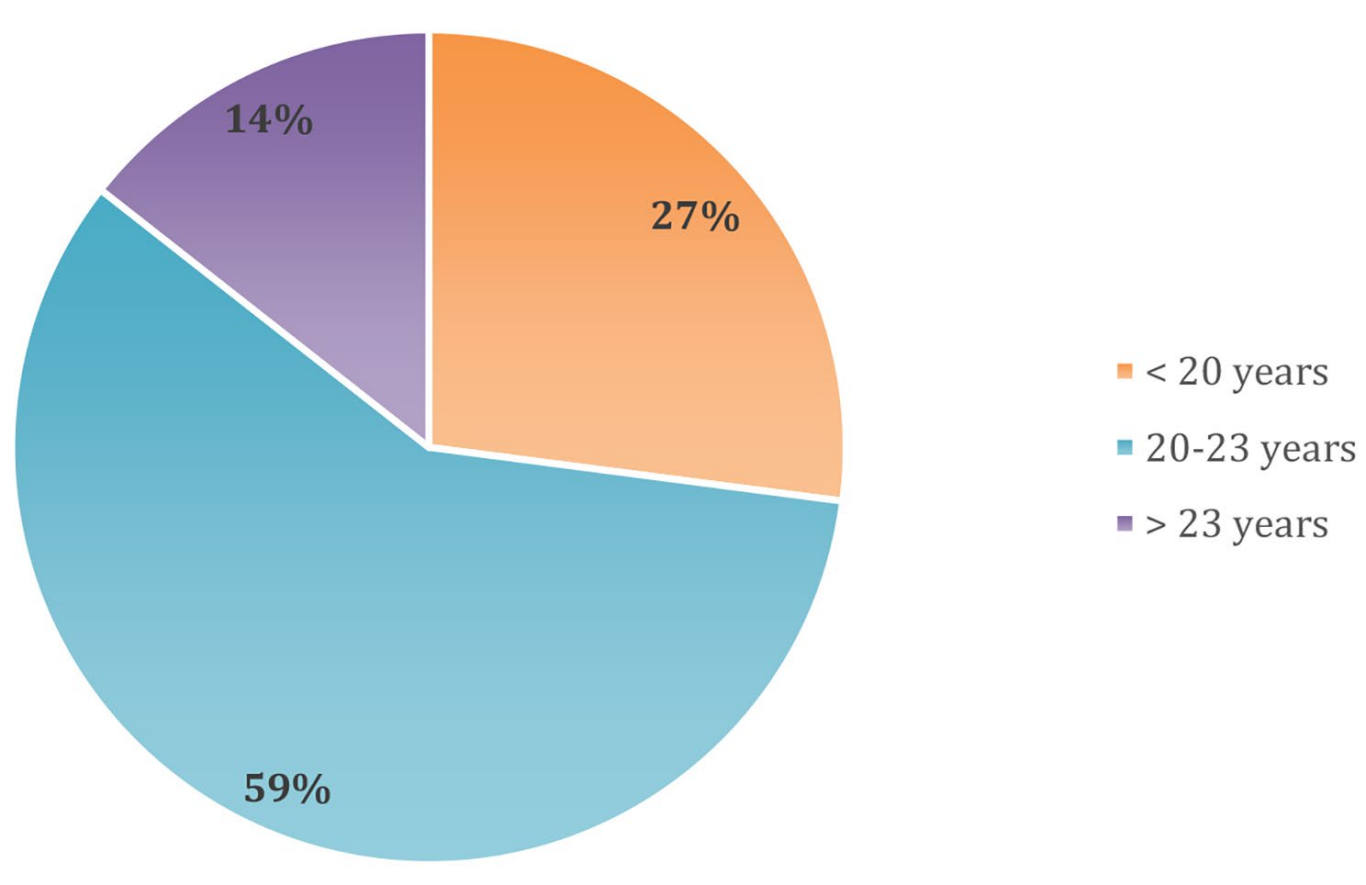
Age groups of the study participants

A total of 350 participants were selected, 99.4% (*N* = 340) agreed to participate in the study and 10 participants who refused to participate were excluded from the analysis. Among the participants, 36.8% (*N* = 125) were males and 63.2% (*N* = 215) were females. The mean age was 21.1 ± 2.1 SD, with a minimum age of 17 years old and a maximum of 27 years old, with 27.1% being < 20 years old, 58.5% between 20 and 23 years, and 14.4% being > 23 years old (Fig. 2). 75% of the participants were admitted to the university through public admission, while 25% were admitted on private funding. Of the 340 participants, only 1.8% (*N* = 6) were married, while the rest (*N* = 334) were single. 71.8% of the participants (*N* = 244) were in the preclinical years, and 28.2% (*N* = 96) were in the clinical years (5th and 6th years) (Table 1). 89.7% (*N* = 305) of the participants were residents of urban areas before admission to the university, while 10.3% (*N* = 35) were residents of rural areas. After admission, 80.3% (*N* = 273) resided with their family or a relative, 15% (*N* = 51) in the university dormitory, 2.6% (N = a 9) in the private dormitory, and 2.1% (*N* = 7) with a friend.

**Table 1:** Sociodemographic criteria of the participants

Variables	N (%)
**Gender**	
Male	125 (36.8)
Female	215 (63.2)
**Marital Status**	
Married	6 (1.8)
Single	336 (98.2)
**College year**	
1st year	54 (15.9)
2nd year	43 (12.6)
3rd year	96 (28.2)
4th year	51 (15)
5th year	45 (13.2)
6th year	51 (15)
**Weekly expenditure**	
< 10,000 SDGs	74 (21.8)
10,000–25,000 SDGs	196 (57.6)
More than 25,000 SDGs	70 (20.6)
**Religion**	
Muslim	334 (98.2)
Atheist/Agnostic/None	6 (1.8)

With regards to weekly expenditure, 57.6% (*N* = 196) spent between 10,000 and 25,000 SDGs, with 21.8% and 20.6% spent less than 10,000 SDGs and more than 25,000 SDGs, respectively (Table 1). The source of income was from parents or a relative in 95.3% (*N* = 324), 3.2% (*N* = 11) from own earnings, and 1.2% (*N* = 4) from the scholarship. The majority of the participants 98.2% (*N* = 334) were Muslims, while 1.8% (*N* = 6) were atheists or agnostic (Table 1).

### Generalized anxiety disorder prevalence

**Fig. 3:**
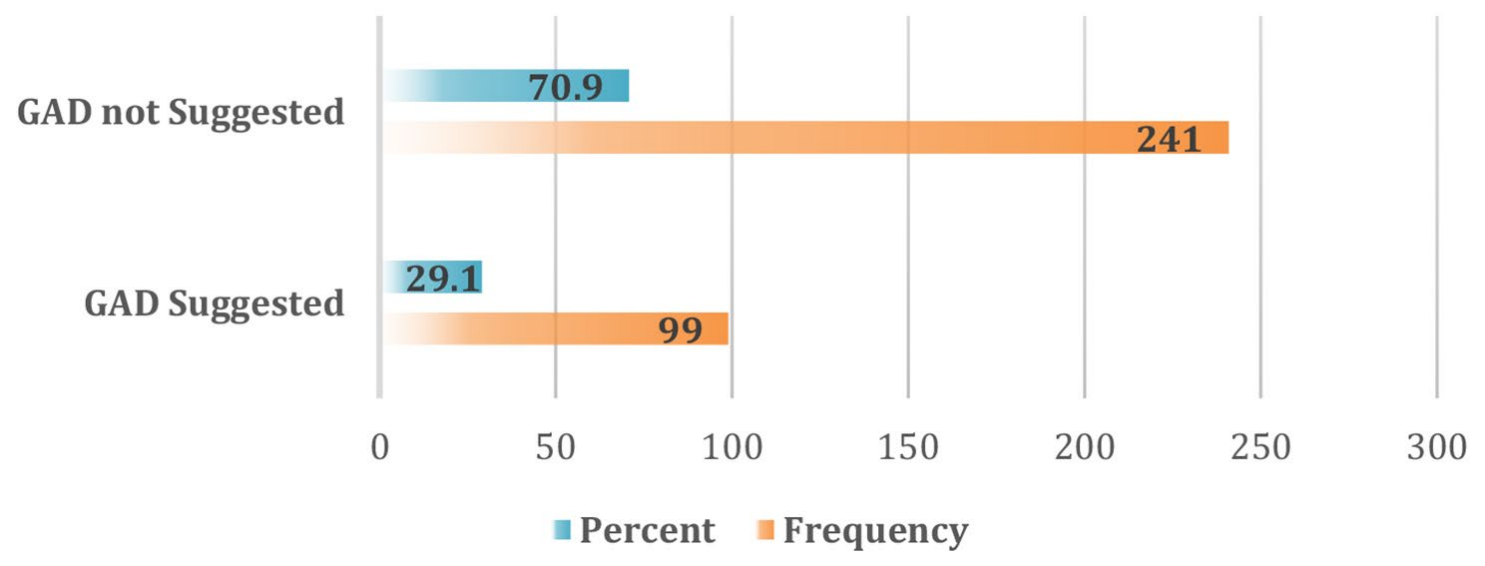
Prevalence of GAD among Sudanese medical students using the GAD-2 scale

Out of 340 participants, only 13 participants (3.8%) were previously diagnosed with GAD by a psychiatrist. By contrast, according to the GAD-2 score, from the total population, 99 participants (29.1%) Scored ≥ 3 in GAD-2 indicating the possibility of developing GAD (Fig. 3). Of these, 7 were diagnosed previously. 6 participants who were diagnosed with GAD previously had scores < 3 (Table 2).

**Table 2:** Reported psychiatric disorders and chronic diseases by the participants

Reported Morbidity	N (%)
**Psychiatric disorders and Chronic Illnesses**	**27 (7.9)**
Depression	10
Bipolar disorder	1
PTSD / Acute stress reaction	1
Personality Disorder	2
IBS	2
Hypothyroidism	1
Asthma	5
SLE	1
Lactose Intolerance	1
**Addictive substances Used (Frequencies)**	**33 (9.7)**
Tobacco/Cigarettes/Vape/Shisha	25
Crystal Methamphetamine (ICE)	5
Cannabis	4
Alcohol	1

Of the 13 participants who had a previous diagnosis of GAD, 3 of them were prescribed Antidepressants only, 2 used relaxation techniques only, 2 received CBT only, 3 used both relaxation techniques and lifestyle modifications as therapy. 2 participants diagnosed with GAD were prescribed combination therapy, Antidepressant and Benzodiazepines in one participant, Antidepressant and B-blocker in the other. To note, one participant was using SSRIs for relieving symptoms despite not seeking a professional diagnosis, and he/she turned out to have a high GAD-2 score. Out of the 13 participants diagnosed with GAD, 8 were having regular follow-ups with a psychiatrist or a psychologist.

### Perceived stress scores

**Fig. 4:**
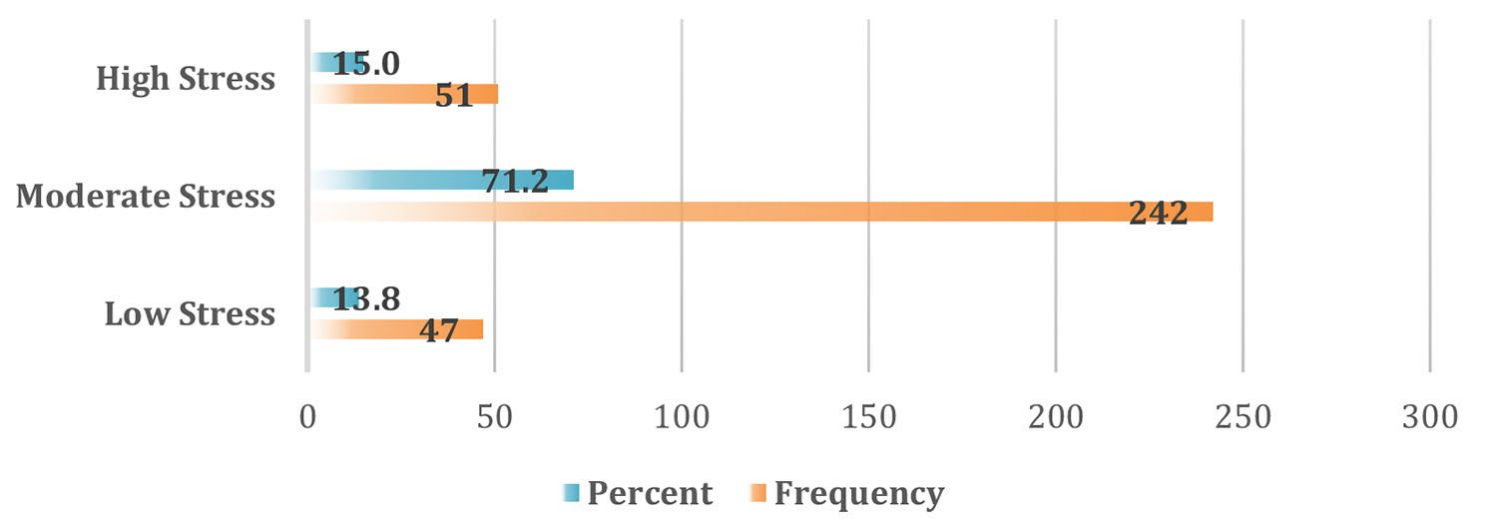
Levels of perceived stress among the participants

On the perceived stress scale, 242 participants (71.2%) appeared to have moderate stress levels, while 47 (13.8%) and 51 (15%) participants had low and high stress levels, respectively (Fig. 4). The mean score was 19.9 ± 6.15 SD.

### Psychiatric disorders, chronic illnesses, and family history

A total of 27 participants stated they had other illnesses. In 13 participants they were psychiatric (depression in 9 participants, bipolar disorder in 1, PTSD, Acute Stress reaction and depression in 1 participant, and personality disorder in 2 participants), 5 of the patients who were previously diagnosed with GAD also had a diagnosis of depression, while 3 patients who had a suggestive GAD score had a previous diagnosis of depression (Table 2). A total of 79 (23%) participants had a family history of psychiatric illness.

### The use of addictive substances

About 23 participants indicated they use Tobacco/Cigarettes or shisha (Hookah) with 9 of them having a suggestive GAD score. 5 indicated the use of Methamphetamine with 3 having a suggestive GAD score. Another 2 use Cannabis along with tobacco and cigarettes, and 2 use Cannabis only (Table 2). Of the 33 participants using addictive substances, 42.4% (*N* = 14) have a high (≥ 3) GAD-2 score.

### Academic performance

The mean academic score for all participants was 2.63 ± 0.84 SD, with a slightly higher mean for the preclinical years’ participants (2.68 ± 0.86 SD) compared to their clinical years’ counterparts (2.51 ± 0.77 SD). Approximately 40% of the participants were unsatisfied with their academic performance, but nearly 30% were satisfied (Table 3).

One-way Analysis of variance (ANOVA) was done to assess the relationship between academic scores and academic performance satisfaction levels. A high mean academic score was found to be associated with higher satisfaction [F(4, 308.29) = 19.3, *p*-value = 0.00)].

### Lifestyle characteristics

Of the 340 participants, 70.6% had unhealthy eating habits, while 29.4% had healthy eating habits. Nearly half of the study participants (47.6%) use smart devices for entertainment purposes between 2 and 4 h daily, while 30% use these devices for the same purpose for more than 6 h per day. 90.3% of the students have enough night sleep (between 4 and 9 h), with only 2.4% (*N* = 8) sleeping less than 4 h per day. Approximately half of the participants (49.1%) indicated that they have acceptable sleep quality, while 28.8% said that they enjoy a good quality of sleep and 22.1% (*N* = 59) indicated poor or unsatisfactory sleep quality. Details regarding other factors are shown in (Table 3).

**Table 3:** Lifestyle characteristics of the study participants

Lifestyle variable	N (%)
**Tea Consumption**	
Never	27 (7.9)
Occasionally	110 (32.4)
Once daily	119 (35.0)
2 times or more	84 (24.7)
**Coffee Consumption**	
Never	80 (23.5)
Occasionally	150 (44.1)
Once daily	73 (21.5)
2 times or more	37 (10.9)
**Energy Beverages Consumption**	
Never	223 (65.6)
Occasionally	94 (27.6)
Once daily	16 (4.7)
2 times or more	7 (2.1)
**Exercise**	
None	148 (43.5)
1 per month	66 (19.4)
> 1/month	53 (15.6)
> 2 times/ week	73 (21.5)
**Eating Habits**	
Unhealthy	57 (16.8)
Not very healthy	183 (53.8)
Rather healthy	90 (26.5)
Very healthy	10 (2.9)
**Hobbies/leisure activities**	
None	45 (13.2)
Rarely	135(39.7)
Occasionally	116 (34.1)
Regularly	44 (12.9)
**Use of smart devices for entertainment**	
< 2 h daily	19 (5.6)
2–4 h daily	162 (47.6)
4–6 h daily	57 (16.8)
> 6 h daily	102 (30.0)
**Sleep Hours**	
< 4 h daily	8 (2.4)
4–6 h daily	117 (34.4)
6–9 h daily	190 (55.9)
> 9 h daily	25 (7.4)
**Sleep Quality**	
Poor	19 (5.6)
Unsatisfactory	56 (16.5)
Acceptable	167 (49.1)
Good	98 (28.8)
**Satisfaction with religious practice**	
Poor	29 (8.6)
Unsatisfactory	53 (15.7)
Acceptable	153 (45.3)
Good	103 (30.5)

### The relationship between GAD, perceived stress, sociodemographic and lifestyle factors

A higher anxiety level was found to be associated with high-stress scores (*p* = 0.000), and this was considered a strong association (*r* = 0.65). No statistical association was found between age and GAD-2 scale score (*p* = 0.106). With Regard to the relationship between weekly expenditure and GAD-2 scale score, a statistically significant difference between the three expenditure groups (< 10,000 SDG, 10,000–25,000 SDG, and > 25,000 SDG) was found (*P* = 0.004, df = 2). No difference in the GAD-2 scale score was observed between the second and third groups.

No statistical association was found between the GAD-2 score and the participants’ mean academic score (*p*-value = 0.179). There was no statistically significant difference in the mean scores for GAD-2 between clinical and preclinical years (*p* = 0.434).

A high GAD score was found to be associated with lower academic performance satisfaction levels (*p*-value = 0.000, df = 4). In post hoc analysis, the difference in scores was found to be between: the fully satisfied-unsatisfied (*p* = 0.004), the fairly satisfied-unsatisfied (*p* = 0.000), and the neutral-unsatisfied (*p* = 0.013).

The Kruskal-Wallis test was done to assess the impact of lifestyle factors on the GAD scale score. low GAD scale score was found to be associated with improved quality of sleep (*p*-value = 0.00, df = 3), satisfaction with religious practices (*p*-value = 0.00, df = 3), and increased time spent on leisure or hobbies (*p*-value = 0.018, df = 3). A High GAD-2 scale score was also found to be associated with increased hours of use of smart devices for entertainment (*p*-value = 0.004, df = 3) and consuming unhealthy foods (*p*-value = 0.004, df = 3). When the same groups are re-organized into two groups (Healthy/Unhealthy), a significant difference in the GAD-2 scores was found between the two groups using Man-Whitney U test (*p* = 0.001, U = 9,322.5, Z= -3.31, *r* = 0.17). For other lifestyle factors, no statistical association was present (Table 4).

**Table 4:** The association between GAD, sociodemographic and lifestyle factors using the Kruskal Wallis and Man-Whitney tests

Variable	GAD Suggested (N)	GAD not Suggested (N)	Total	P-value
**Gender**				
Male Female Total	35 64 99	90 151 241	125 215 340	0.649
**College Year**				
Preclinical Clinical Total	75 24 99	169 72 241	244 96 340	0.434
**Weekly expenditure**				
< 10000 SDG 10000–25000 SDG > 25000 SDG Total	10 68 21 99	64 128 49 241	74 196 70 340	0.004*
**Academic performance satisfaction levels**				
Unsatisfied Fairly unsatisfied Neutral Fairly Satisfied Fully satisfied Total	33 21 27 12 6 99	33 55 69 60 24 241	66 76 96 72 30 340	< 0.000*
**Quality of sleep**				
Poor Unsatisfactory Acceptable Good Total	15 25 43 16 99	4 31 124 82 241	19 56 167 98 340	< 0.000*
**Satisfaction with religious practices**				
Poor Unsatisfactory Acceptable Good Total	12 20 48 18 98	17 33 105 85 240	29 53 153 103 338	< 0.000*
**Use of smart devices for entertainment**				
< 2 h daily 2–4 h daily 4–6 h daily > 6 h daily Total	3 45 21 30 99	16 117 36 72 241	19 162 57 102 340	0.004*
**Eating Habits**				
Unhealthy Not very healthy Rather healthy Very healthy Total	22 55 20 2 99	35 128 70 8 241	57 183 90 10 340	0.004*

### The association between perceived stress, sociodemographic and lifestyle

One-way Analysis of variance (ANOVA) was done to assess the relationship between age and PSS. It was found that age > 23 is associated with less PSS score as compared to age < 23 (*p* = 0.035). Post hoc analysis resulted in [F (2,132.9) = 3.34, *p*-value = 0.038]. The same relationship was confirmed by a one-tailed Pearson correlation coefficient (*p* = 0.035, Coefficient = − 0.098). Having a family history of psychiatric illness was found to be associated with increased PSS scores (*p* = 0.016, the mean difference is 2.1 ± 0.88 SD).

No statistical association was found between perceived stress score and participants’ mean academic score (*p*-value = 0.770). There was no statistically significant difference in the mean scores for perceived stress between clinical and preclinical years (*p* = 0.56). A higher stress level was found to be associated with lower academic performance satisfaction levels [F (4, 240,9) = 8.7, *p*-value = 0.00). Since academic performance satisfaction was found to be positively associated with academic scores (*p*-value = 0.00), this makes academic scoring an indirect modulator for anxiety and perceived stress among medical students.

One-way Analysis of variance (ANOVA) was done to assess the impact of lifestyle factors on perceived stress levels. Reduced levels of perceived stress were found to be associated with improved quality of sleep [F(3, 127,4) = 15.1, *p*-value = 0.00)], satisfaction with religious practices [F(3, 166.6) = 10.8, *p*-value = 0.00)], increased time spent on leisure or hobbies [F(3, 195.9) = 5.2, *p*-value = 0.002)], having a healthier diet [F(3,45.6.5) = 4.6, *p*-value = 0.006)]. High Perceived stress scores were associated with increased hours of use of smart devices for entertainment [F(3, 128.5) = 6.2, *p*-value = 0.001)]. For other lifestyle factors, no statistical association was present (Table 5).

**Table 5:** The association between perceived stress, sociodemographic and lifestyle factors using Analysis of variance (ANOVA) test

Variable	Low stress	Moderate Stress	High Stress	Total	P-value
**Age**					
< 20 years 20–23 years > 23 years Total	5 28 14 47	78 135 29 242	9 36 9 51	92 199 49 340	0.035*
**College Year**					
Preclinical Clinical Total	29 18 47	183 59 242	32 19 51	244 96 340	0.56
**Weekly expenditure**					
< 10000 SDG 10000–25000 SDG > 25000 SDG Total	8 26 13 47	59 136 47 242	7 34 10 51	74 196 70 340	0.387
**Academic performance satisfaction levels**					
Unsatisfied Fairly unsatisfied Neutral Fairly satisfied Fully satisfied Total	4 9 15 13 6 47	43 55 67 55 22 242	19 12 14 4 2 51	66 76 96 72 30 340	0.000*
**Quality of sleep**					
Poor Unsatisfactory Acceptable Good Total	0 4 15 28 47	11 41 129 61 242	8 11 23 9 51	19 56 167 98 340	0.000*
**Satisfaction with religious practices**					
Poor Unsatisfactory Acceptable Good Total	1 5 13 28 47	21 34 119 67 241	7 14 21 8 50	29 53 153 103 338	0.000*
**Use of smart devices for entertainment**					
< 2 h daily 2–4 h daily 4–6 h daily > 6 h daily Total	7 19 5 16 47	11 125 41 65 242	1 18 11 21 51	19 162 57 102 340	0.001*
**Eating Habits**					
Unhealthy Not very healthy Rather healthy Very healthy Total	7 22 16 2 47	35 134 67 6 242	15 27 7 2 51	57 183 90 10 340	0.006*

## Discussion

Our research sociodemographic data showed that 85.6% of students were < 23 years old, mostly single, nearly two-thirds of participants were females, and only a quarter of participants were admitted to the university by private funding. Nine out of 10 students were living in urban areas in the country before entering university (89.7%). Nearly 20% of the participants who scored high on the GAD-2 scale are spending less than 10 thousand Sudanese pounds/per week (around 18 USD/week and 2.4 USD/day). Several studies talked about the psychological distress associated with the financial burden on medical students [27–29]. Studies conducted in China and Germany showed that financial difficulty is associated with anxiety, and psychosomatic symptoms [30, 31]. A study in twenty-two Brazilian medical schools found that financial aid and scholarship students experienced less state anxiety but not trait anxiety [32]. To the best of the authors’ knowledge, there is no data about the effect of inflation and progressive loss of purchasing power of the Sudanese pound on the mental health of medical students in Sudan but keeping in mind that this expenditure is almost equal to the new world bank extreme poverty line in September 2022 [33].

In our study, 3.8% of the students already had a previous psychiatric diagnosis of Generalized Anxiety disorder, corresponding to the frequency of GAD clinically in US adults 4–7% [34, 35]. However, nearly one-third of the students scored high in GAD-2 (GAD-2 score ≥ 3) indicating the probability of having GAD. This discrepancy can be attributed to the decreased exposure of the students to mental health programs and advocacies, inefficient mental health services, and poor accessibility as well as the stigma of visiting mental health professionals or seeking help through psychiatrists or psychologists. This was supported by a 2018 study at Khartoum University which proved that more than half of medical students declared that what abstain them from psychological counseling were the feeling the discrimination, believing in their competency to deal with their problems, apprehension of the unfamiliar and failing to identify symptoms at 63%, 60%, 59%, and 58% respectively [29]. Equivalently to our study, Asian students suffered from high levels of perceived stress, poor habits, financial restrictions, and deficiencies in exercise and joyful programs, thus exposing students to anxiety and lower quality of Life [10].

A Sudanese study carried out at the same university in 2016, stated that 22% and 16% of medical students had moderate degrees of anxiety and stress respectively, using the depression, anxiety, and stress scale (DASS 21) [19]. Comparing that to the global prevalence of anxiety among medical students of 33.8% which was most prevalent among Middle Eastern and Asian students [18].

Among the 13 participants who were already diagnosed with GAD, five were also diagnosed with depression, while 3 students who had a suggestive GAD score had a diagnosis of depression as it is a predictable comorbidity [4, 36]. The comorbidity rates of anxiety cases with depression reaching 19.2% have been reported [2]. Moreover, the National Comorbidity Studies (NCS) found that 14% of the population had three or more comorbid psychiatric disorders [28].

In our study, high perceived stress was associated with having a family history of psychiatric illness, it is well known that stress can be a precipitating factor for developing psychiatric illnesses, especially among those genetically predisposed [1, 4]. Family history is a well-known risk factor that can affect not only the incidence of the disorder but also its prognosis and course as well, through genetic elements, and also by sharing the same environmental risk factors [37, 38].

A high GAD-2 score was strongly associated with perceived stress; this is in line with the literature that suggests that perceived stress is a predictor of anxiety. A study among 1233 medical workers from three hospitals in China using a perceived stress questionnaire (PSQ) associated perceived stress with anxiety and depression and suggested it might have a mediating effect. Another study among nursing students in China found a strong association between perceived stress and anxiety [39, 40].

We found that most of the students had moderate stress levels (71.2%). low and elevated stress levels were almost similar at 13.8% and 15% respectively. Medical students experience higher levels of stress as compared to their peers; this was demonstrated in a study on Egyptian medical students with 63% of participants reporting symptoms of stress [41]. Furthermore, compared to our study, severe stress rates in Saudi undergraduate healthcare students were lower by 2.3% [42].

Similarly, some international studies showed comparable results to our study concerning moderate stress levels [43, 44]. The moderate stress levels in the Sudanese medical students at Khartoum University have increased by 55% since 2016 (from 16 to 71.2%) keeping in mind the former study used DASS21 as a measurement for stress [19]. A relatively lower Stress level of 44% was spotted in Ethiopian medical students [45]. Medical students are exposed to different stressors such as examinations, as provided in a 2014 study in Morocco [46]. Furthermore, a 2019 longitudinal study showed the leading stressors as specified by the students were money matters, nonacademic life aspects, individual’s competency/stamina, as well as syllabus/surroundings [46]. Other causes proved by WHO-ICI surveys “in between two-thirds and three-fourths of participants experienced at least mild stress about problems facing their loved ones (74.8%), their financial status (68.6%), intimate relationships (66.8%), and their well-being (64.3%). About half of the respondents experienced at least mild stress about their family’s connection (56.7%) and communication with students or colleagues (52.9%)” [47]. Over time, chronic stress can lead to burnout impacting the students’ cognition and intelligence destructively [48, 49].

We found that the mean academic score of the students was neither associated with high GAD-2 scores nor it was with high perceived stress, but interestingly lower academic performance satisfaction level was associated with high GAD-2 score (*p*-value = 0.000), and high PSS scores (*p*-value = 0.00). Also, the mean academic score was positively associated with academic performance satisfaction (*p*-value = 0.00), making the mean academic score an indirect modulator for anxiety and perceived stress. This positive association between the mean academic score and academic performance satisfaction corresponded with other health professions students [50, 51].

These results we obtained are consistent with the nature of Generalized anxiety disorder (GAD) as individuals diagnosed with GAD have low self-esteem, worry excessively about everyday life, and feel like they cannot control their worry so it is a possibility that this anxiety or worry about the academics is surfacing as being unsatisfied with the academic performance. A similar result to ours was found in a longitudinal study about perceived stress among chiropractic students in Iowa in which pre-matriculation GPA was not a predictor of perceived stress [52]. In contrast, a study in Malaysia established a weak and negative relationship between stress and academic achievement [53]. A study about stress, burnout, and associated risk factors in Saudi Arabian medical students showed students with low GPAs experienced higher stress, burnout, and emotional exhaustion [54]. Kötter and Wagner used the “Perceived Medical School Stress Instrument PMSS” and found that higher scores predicted the students’ performance in their first medical examination [55]. In our results, mean academic score is not directly associated with perceived stress, this can be attributed to the fact that at the faculty of medicine at the University of Khartoum, GPA is not offered directly; rather an individual grading for each subject is done, nevertheless, this is an area that still needs further research.

There was no significant difference in mean stress levels between preclinical and clinical students consistent with previous literature [32, 56]. In contrast; many international studies have come to diverse results, regarding preclinical and clinical years in comparison to stress and burnout [46, 57, 58]. A qualitative study in Birmingham highlighted those transitional periods, approaching qualification, and acquiring skills were reported as stressful [59]. Another study in Turkey about anxieties of clinical training from 2 medical schools that adopted 2 different curricular models concluded that students who were exposed to clinical skills didn’t have much anxiety later on because of the smooth transition from basic to clinical sciences although they experienced anxiety regarding their communication skills [60]. Also, the constant stress and anxiety from the rich medical syllabus followed by continuous examinations, fear of failure, pressure to pass, and peer competitiveness, as well as adapting to new environments, socializing, and often financial limitations, all affect a student’s quality of life [61].

We found that good sleep quality is associated with low GAD-2 score and low perceived stress, as it is well established that lack of sleep is associated with more stress levels, students who have insomnia have more stress and anxiety symptoms [14, 62]. A study in Saudi Arabia strongly associated stress with poor sleep quality and found that students who were less stressed were less likely to experience poor sleep quality [62]. In addition, students with lower GPAs deprive themselves of sleep, to improve their grades, affecting their mental health negatively [49, 63]. Poor sleep quality and Sleep deprivation are prevalent in medical students [64–66].

Our study sheds light on students’ overindulging unhealthy eating habits at 70.6%, who were found to have high GAD-2 scores. Whereas, adopting a healthy diet was found to be associated with lower perceived stress levels. A cross-sectional study involving students from 7 countries found that increased stress leads to maladaptive eating behaviors [67]. Corresponding to previous studies, widespread unhealthy eating habits such as skipping breakfast and infrequent daily meals were the most frequent unhealthy habits associated with stress in Iraqi medical students (60.4% and 56.7% respectively) [68]. Most Indian medical students consume fast and fried food. Similarly, many unhealthy behaviors have been identified to be associated with increased stress such as infrequent exercise, alcohol drinking, smoking, sleep disorders, and eating poorly [69–71].

Only 6.8% of medical students at Khartoum University use cigarettes/tobacco or shisha (Hookah) which is lower by 3.8% of cigarette smoking prevalence among medical students at National Ribat University in Sudan [72]. Only 2% used marijuana or abused methamphetamine. An international meta-analysis concluded 1 in 3 medical students has used cannabis, whereas 8.8% were current users [73].

As expected, excessive smart device use for entertainment i.e., ≥ 2 h daily, was noticed in more than three-quarters of the study sample. A high magnitude of smartphone usage and addiction by medical students correlated with poorer sleep quality and perceived stress [74, 75]. Our study found an association between the increased hours of smart devices used for entertainment and high GAD-2 scores as well as high perceived stress. A systematic review and meta-analysis study concluded that problematic smartphone usage was associated with increased odds of anxiety (OR = 3.05; 95%CI; 2.64–3.53; I 2 = 0%) and perceived stress (OR = 1.86;95%CI 1.24–2.77; I 2 = 65%) [76]. Also, we found that low GAD-2 scores and low perceived stress were associated with increased time spent on leisure or hobbies. As it is well established now that exercise can help reduce anxiety levels [67, 77], it is understandable that students who can find time to practice their hobbies or exercise have lower levels of anxiety.

Three-quarters of the students were content with their religious practice which is a well-known coping mechanism if used positively. In a small focus group (*n* = 4) in New Zealand, students believed that having a belief system assisted them when coping with the academic learning environment [78]. In Iran, they revealed that the sound of the Quran before exams can reduce students’ anxiety levels which highlights the satisfaction with religious practices associated with low GAD-2 scores in this study [79].

### Strengths and limitations

This is the first study among Sudanese medical students to explore the association of perceived stress and Generalized anxiety disorder (GAD), it is also among the few in the country that studied perceived stress among medical or even university students and its relationship with academic performance and lifestyle factors. The strength of this study comes from its consideration of different factors that may cause or contribute to GAD and increased perceived stress. Nevertheless, being a cross-sectional study limits the conclusiveness of some results that need longitudinal or qualitative studies, namely the relation between academic performance portrayed by mean academic score and high perceived stress. The study was conducted at the Faculty of Medicine - University of Khartoum, although it is a large campus with diversity in its socio-demographics the results cannot be generalized to all medical students in Khartoum.

### Recommendations

Several interventions are necessary to mitigate the effects of anxiety and perceived stress among medical students, widening access to mental health services and providing safe channels for students can allow early diagnosis of GAD and help students manage their stress by providing health education about coping with stress and promoting a healthy lifestyle. Safe channels can be in the form of a hotline or a website offering confidential counseling or organizing and supervising online support groups. The faculty can also consider integrating health education about the same topics into the curriculum and should support the existing students’ initiatives. These initiatives can organize support groups and promote self-care habits [80], meditation, and any other proven strategies to tackle stress and anxiety. Further research should be focused on the effect of different factors like curricular elements, differences in university grading systems, and other factors that may have caused the difference between studies.

Other universities should also endorse and encourage establishing students’ initiatives if not already existing. Procedures regarding close student follow-up can be beneficial, several universities apply for academic supervision programs in which a member of the faculty staff closely follows a small group of students to help with their problems, such procedures if correctly implemented can help students in several aspects including effective learning, efficient studying techniques and can aid in early detection and referral of students in need to proper mental health care service. Providing access to diverse activities within the medical school encourages students to explore and practice their hobbies as it was found to be associated with low levels of perceived stress and GAD-2 scales. Policies about supporting students financially should be activated and initiated if not existing, this may exceed the faculty level and urges a discussion and concerns to be raised to different stakeholders of the medical education sector. Lastly, future research on web-based interventions is recommended to investigate and embrace healthier lifestyles [81–83].

## Conclusion

Among our study population, few participants were diagnosed with GAD by a psychiatrist, 29.1% of participants scored high on the GAD-2 (score ≥ 3), a finding that encourages the establishment of periodic mental health screening programs for medical students, early diagnosis of high-risk individuals, and early interventions through confidential access to mental health services. Anxiety levels and perceived stress were strongly and positively associated. We found that students who spent less than 10,000 SDG/weekly scored high on the GAD-2 scale, indicating the impact of the financial burden on medical students. The percentage of substance use in the study was 9.7%, with 42% of them having a high GAD score, which requires more investigation and early intervention to prevent further harm to the student’s health. It was also found that a high GAD score was associated with the consumption of unhealthy food, increased hours of smart devices used for entertainment, and low quality of sleep, which highlights several modifiable risk factors of GAD. Increased perceived stress level was found to be associated with lower academic performance satisfaction levels. It was found that age > 23 is associated with a lower perceived stress score.

## Data Availability

The dataset used and analyzed during this study is available from the corresponding author upon reasonable request.

## Abbreviations

ANOVA: Analysis of variance
CBT: Cognitive behavioral therapy
DASS21: Depression, anxiety, and stress scale- 21 items
DSM-V-TR: Diagnostic and statistical manual text revision fifth edition
DSM-VI: Diagnostic and Statistical Manual Fourth edition
GAD: Generalized Anxiety Disorder
GAD-2: Generalized Anxiety Disorder- 2-item scale
GPA: Grade point average
ICE: Crystal Methamphetamine
IBS: Irritable bowel disease
SDG: Sudanese pound
SPSS: Statistical package of social sciences
SSRI: Selective serotonin reuptake inhibitor
SLE: Systemic Lupus Erythematosus
PTSD: Post-traumatic stress disorder
PRIME-MD-PHQ: Primary Care Evaluation of Mental Disorders Patient Health Questionnaire
PMSS: Perceived Medical School Stress Instrument
PSS-10: Perceived stress − 10 items scale
PSQ: Perceived stress questionnaire
WMHS: World Mental Health Surveys
WHO-ICI: World Health Organization- International College Students initiative
